# Thermal Analysis of Binary Mixtures of Imidazolium, Pyridinium, Pyrrolidinium, and Piperidinium Ionic Liquids

**DOI:** 10.3390/molecules26216383

**Published:** 2021-10-22

**Authors:** Elena Gómez, Pedro Velho, Ángeles Domínguez, Eugénia A. Macedo

**Affiliations:** 1 Associate Laboratory of Separation and Reaction Engineering–Laboratory of Catalysis and Materials (LSRE-LCM), Department of Chemical Engineering, Faculty of Engineering, University of Porto, 4200-465 Porto, Portugal; elenacostas@fe.up.pt (E.G.); velho@fe.up.pt (P.V.); 2 Advanced Separation Processes Group, Department of Chemical Engineering, University of Vigo, Campus Lagoas-Marcosende, 36310 Vigo, Spain; admguez@uvigo.es

**Keywords:** ionic liquids, heat capacity, thermal behavior, heat capacity, DSC

## Abstract

Ionic liquids (ILs) are being widely studied due to their unique properties, which make them potential candidates for conventional solvents. To study whether binary mixtures of pure ionic liquids provide a viable alternative to pure ionic liquids for different applications, in this work, the thermal analysis and molar heat capacities of five equimolar binary mixtures of ionic liquids based on imidazolium, pyridinium, pyrrolidinium, and piperidinium cations with dicyanamide, trifluoromethanesulfonate, and bis(trifluoromethylsulfonyl)imide anions have been performed. Furthermore, two pure ionic liquids based on piperidinium cation have been thermally characterized and the heat capacity of one of them has been measured. The determination and evaluation of both the transition temperatures and the molar heat capacities was carried out using differential scanning calorimetry (DSC). It was observed that the thermal behavior of the mixtures was completely different than the thermal behavior of the pure ionic liquids present, while the molar heat capacities of the binary mixtures were very similar to the value of the average of molar heat capacities of the two pure ionic liquids.

## 1. Introduction

In recent years, both the thermophysical properties and the application of ionic liquids (ILs) in different processes have been widely studied. This high interest in these compounds is mainly due to their special characteristics such as low vapor pressure and their high solvent power for a wide variety of chemical compounds, both polar and nonpolar. Moreover, they are liquid in a wide range of temperatures, which makes them more versatile.

One of the most important properties of ionic liquids is their so-called “tunability”, i.e., the possibility of synthesizing a suitable ionic liquid for a determined application. Within this approach, the possibility of mixing ionic liquids is a viable alternative, since mixed ILs can offer a significant improvement in the phase behavior properties and extend their applicability to other processes. There are many studies on the thermal analysis of pure ILs, however, there is a lack of studies related to heat capacities and transition temperatures of binary mixtures of ionic liquids in the literature [1,2,3,4,5,6,7].

In this context, with the purpose of calculating and comparing the thermal behavior and the molar heat capacities (Cp) of equimolar mixtures of two ionic liquids with the pure ionic liquids that form the mixture, the transition temperatures and the heat capacities of five equimolar mixtures of ionic liquids will be determined and evaluated using differential scanning calorimetry (DSC). The ionic liquids used in the mixtures were chosen so as to study the influence of the anion of the ionic liquid on the thermal properties of the mixtures. Therefore, the selected mixtures were the following: 1-butyl-3-methylimidazolium bis(trifluoromethylsulfonyl)imide ((1,3) BMimNTf_2_) + 1-butyl-3-methylimidazolium trifluoromehtylsulfonate ((1,3) BMimTFO); 1-butyl-3-methylimidazolium bis(trifluoromethylsulfonyl)imide ((1,3) BMimNTf_2_) + 1-butyl-3-methylimidazolium dicyanamide ((1,3) BMimDCA); 1-butyl-3-methylpyridinium bis(trifluoromethylsulfonyl)imide ((1,3) BMpyNTf_2_) + 1-butyl-3-methylimidazolium trifluoromethanesulfonate ((1,3) BMpyTFO); 1-butyl-1-methylpyrrolidinium bis(trifluoromethylsulfonyl)imide ((1,1) BMpyrNTf_2_) + 1-butyl-1-methylpyrrolidinium trifluoromethanesulfonate ((1,1) BMpyrTFO) and 1-methyl-1-propylpiperidinium bis(trifluoromethylsulfonyl)imide ((1,1) PMpipNTf_2_) + 1-methyl-1-propylpiperidinium trifluoromethanesulfonate ((1,1) PMpipTFO). No results have been found for comparison purposes for these mixtures

Furthermore, the thermal behavior for two the pure ionic liquids 1-methyl-1-propylpiperidinium bis(trifluoromethylsulfonyl)imide and 1-methyl-1-propylpiperidinium trifluoromethanesulfonate and the molar heat capacity as a function of temperature for the 1-methyl-1-propylpiperidinium bis(trifluoromethylsulfonyl)imide were carried out using DSC. The transition temperatures of pure 1-methyl-1-propylpiperidinium bis(trifluoromethylsulfonyl)imide were compared with literature values [8,9]; for the other pure ionic liquid, no values have been found of heat capacities or transition temperatures in the literature.

The transition temperatures of the five equimolar mixtures and the two pure ionic liquids were carried out cooling from 393.15 K to 133.15 K and heating from 133.15 K to 393.15 K at a rate of 2 K·min^−1^. The molar heat capacities for the mixtures were measured for the 258.15–348.15 K, and for the 1-methyl-1-propylpiperidinium bis(trifluoromethylsulfonyl)imide for the 293.15–393.15 K.

## 2. Results and Discussion

### 2.1. Pure Ionic Liquids

#### 2.1.1. Thermal Behavior

Of the nine ionic liquids chosen to form the binary mixtures studied in this work, two of them (1-methyl-1-propylpiperidinium bis(trifluoromethylsulfonyl)imide and 1-methyl-1-propylpiperidinium trifluoromethanesulfonate) were thermally characterized by determining their transition temperatures and their Cp values as a function of temperature.

**Figure 1:**
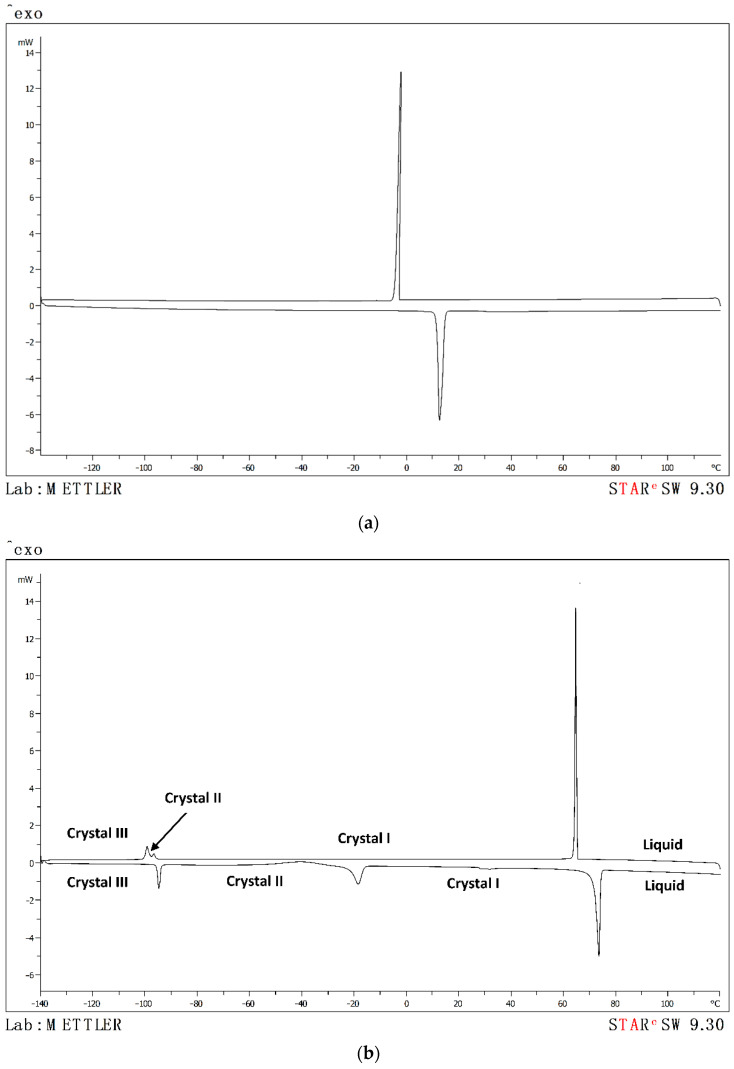
Thermogram cooling the sample from 393.15 K to 133.15 K and heating from 393.15 K to 133.15 K at a rate of 2 K·min^−1^ for pure ionic liquids: (**a**) (1,1) PMpipNTf_2_ and (**b**) (1,1) PMpipTFO.

Table 1 presents the values of the transition temperatures determined with DSC for the pure ionic liquids ((1,1) PMpipNTf_2_ and (1,1) PMpipTFO), together with the values found in the literature [8,9]. Figure 1 shows the respective thermograms.

As can be observed in Table 1 and Figure 1, the two piperidinium-based ionic liquids exhibit freezing and fusion points, therefore these ionic liquids are good crystal formers. The ionic liquid (1,1) PMpipNTf_2_ only presents one freezing and fusion temperature (Figure 1a), while in the thermogram for the ionic liquid (1,1) PMpipTFO (Figure 1b) three peaks can be observed on heating and three peaks on cooling. The first peak on cooling (Tf_(L-I)_) corresponds to fusion, i.e., to the transition of the liquid state to the first solid state (crystal I). The second peak (Tf_(I-II)_) is the change of morphology of crystal I to crystal II, the second solid state. Lastly, the third peak (Tf_(II-III)_) corresponds to the conversion from crystal II to crystal III. Following the same logic, on heating, three peaks appear that could be related to melting points, however, the first peak (Tm_(III-II)_) corresponds to the change of state from crystal III to II, the second Tm_(II-I)_ corresponds to the change of crystal II to crystal I and the last peak (Tm_(I-L)_) is the melting point of this ionic liquid. This behavior is characteristic of polymorphic materials [10].

When comparing the obtained results with data from the literature, also shown in Table 1, the experimental melting point for (1,1) PMpipNTf_2_ is very similar to the bibliography, although the equipment and method used were different. For (1,1) PMpipTFO, no bibliography information was found.

#### 2.1.2. Heat Capacities

**Figure 2:**
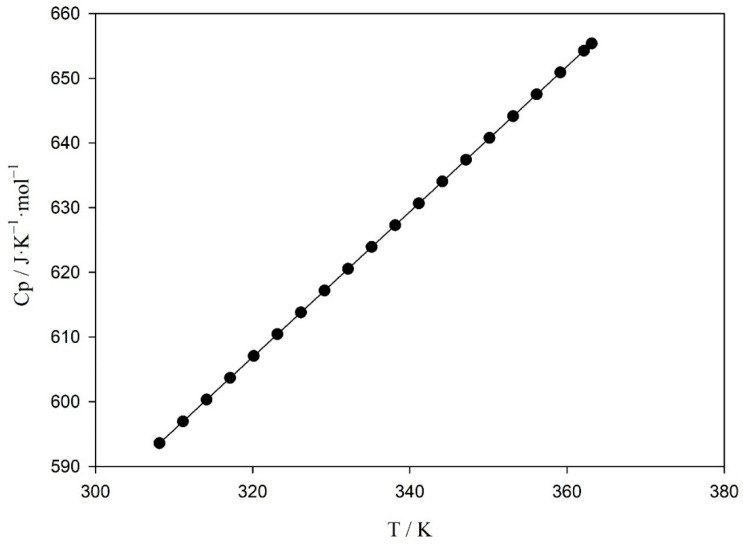
Experimental molar heat capacities, Cp, as a function of temperature for the (1,1) PMpipNTf_2_, solid lines represent the fitting using Equation (1).

Table S1 (available in Supporting Material) and Figure 2 present the experimental molar heat capacities for the pure ionic liquid evaluated in this work in the temperature range 308.15–363.15 K. A linear equation was used for the fitting of the molar heat capacity with temperature:

Cp = *y*_0_ + *a*T (1)

The fitting parameters (*y*_0_, and *a*) of Equation (1) and the correlation factor, R^2^, are shown in Table 2, and the graphical representation of Cp values as a function of temperature and the fitting can be seen in Figure 2. As can be observed from this figure, the heat capacity increases linearly with temperature, which is the expected trend and the common behavior on this property of ionic liquids [10,11,12].

### 2.2. Mixtures of Ionic Liquids

#### 2.2.1. Thermal Behavior

To determine and evaluate the transition temperatures in the DSC, five equimolar mixtures of different pairs of ionic liquids with imidazolium, pyridinium, pyrrolidinium, and piperidinium cations and dicyanamide, trifluoromethanesulfonate and bis(trifluorosulfonyl)imide anions have been prepared. The binary mixtures were: (1,3) BMimTFO + (1,3) BMimNTf_2_; (1,3) BMimDCA + (1,3) BMimNTf_2_; (1,3) BMpyTFO + (1,3) BMpyNTf_2_; (1,1) BMpyrTFO + (1,1) BMpyrNTf_2_ and (1,1) PMpipTFO + (1,1) PMpipNTf_2_. They were selected with the aim of investigating the influence of the anion of the ionic liquid on the thermal behavior.

The values of the transition temperatures determined for the mixtures from thermal analysis and for the pure ionic liquids from previous works for the same group [10,11,12] can be observed in Table 3 in order to ease comparison. It can be concluded that the equimolar binary mixtures of ionic liquids present a totally different thermal behavior than the pure ionic liquids that constitute the mixture, since the mixtures evaluated in this work do not show crystallization in the range of temperatures studied and only present glass transition temperatures.

**Figure 3:**
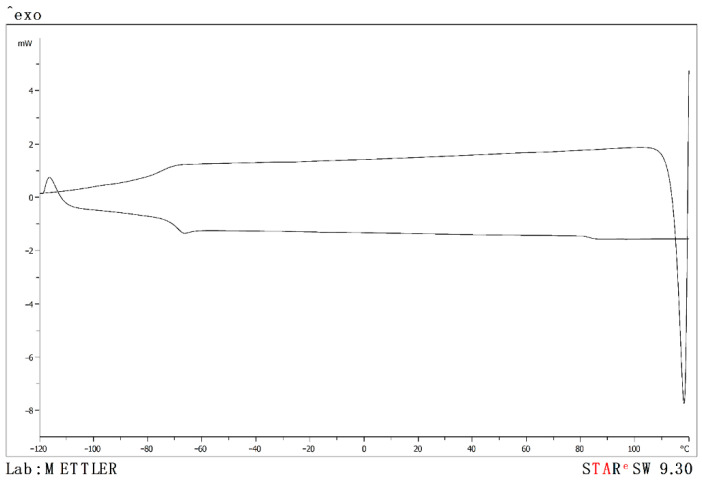
Thermogram cooling the sample from 393.15 K to 133.15 K and heating from 393.15 K to 133.15 K at a rate of 2 K·min^−1^ for the equimolar binary mixture (1,1) PMpipTFO + (1,1) PMpipNTf_2_.

Figure 3 shows, for example, the thermogram for mixture (1,1) PMpipTFO + (1,1) PMpipNTf_2_. The remaining thermograms are available in the Supporting Material (Figure S1).

#### 2.2.2. Heat Capacities

**Figure 4:**
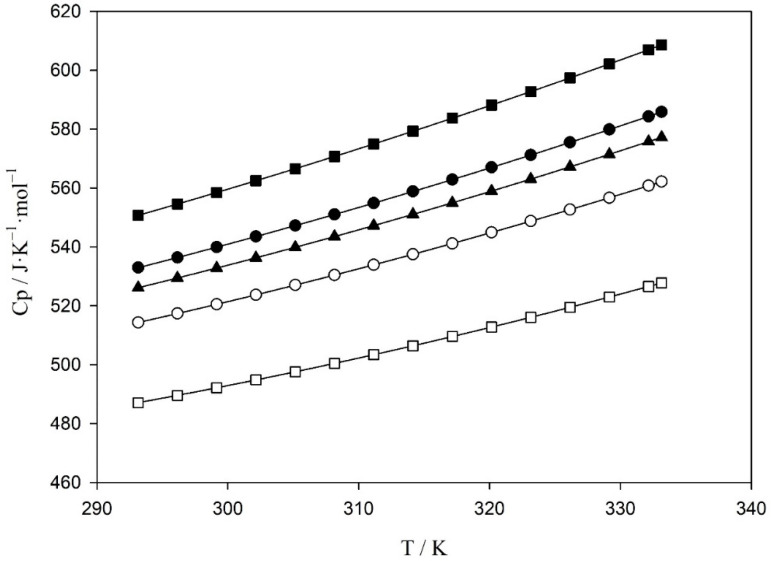
Experimental molar heat capacities, Cp, as a function of temperature for the equimolar binary mixtures: (○) (1,3) BMimTFO + (1,3) BMimNTf_2_; (□) (1,3) BMimDCA + (1,3) BMimNTf_2_; (▲) (1,3) BMpyTFO + (1,3) BMpyNTf_2_; (●) (1,1) BMpyrTFO + (1,1) BMpyrNTf_2_ and (■) (1,1) PMpipTFO + (1,1) PMpipNTf_2_, solid lines represent the fitting using the Equation (2).

The molar heat capacity of the five equimolar binary mixtures of ionic liquids has been determined by DSC with the method of sapphire in the range of 298.15–348.15 K. In Table S2 (Supporting Material) and Figure 4, the experimental molar heat capacities for the mixtures of ionic liquids in the temperature range 293.15–333.15 K are presented.

Table 4 shows the results at 323 K and the Cp of the pure ionic liquids at the same temperature [10,11,12] for comparison purposes. The five binary mixtures showed a value of Cp which is intermediate of the values of the individual ionic liquids, therefore, these Cp values reveal that the Cp of the two pure ionic liquids mixtures exhibits a practically ideal behavior, this behavior was also observed and reported in the bibliography [8] for mixtures of other ionic liquids. Furthermore, it can be seen that the piperidinium-based ionic liquid present higher heat capacity than their analogous pyrrolidinium, pyridinium, and imidazolium-based ionic liquids, in agreement with previous publications on the topic [6].

The fitting of the molar heat capacity of the equimolar mixtures with temperature was carried out using a polynomial equation:

Cp = *y*_0_ + *a*T + *b*T^2^ (2)

Table 5 shows the fitting parameters (*y*_0_, *a*, and *b*) and the correlation factor, R^2^. Moreover, in Figure 4, the fitting is plotted. As can be observed in this figure, the dependence of the Cp of the equimolar mixtures of two ionic liquids with the temperature is practically linear.

## 3. Materials and Methods

### 3.1. Materials

The name, abbreviation used in this work, purity (in mole fraction), and water content of the nine ionic liquids used in this study are specified in Table 6.

The nine ionic liquids were purchased from IoLitec (Heilbroon, Germany) and the water content given in Table 1 corresponds to the amount of water determined after purification (vacuum drying at 343 K and 0.2 Pa for at least 48 h) using a coulometric Karl Fischer titrator, model C20, using Hydranal-Coulomat CG and Hydranal-Coulomat AG as cathodic and anodic titrant. The measurements of water content were carried out prior to the determination of the molar heat capacities and transition temperatures.

### 3.2. Methods

The determination of the thermal behavior and the heat capacities of the pure ionic liquids and their binary mixtures were carried out using a Mettler-Toledo differential scanning calorimeter (DSC) (Mettler-Toledo, OH, USA), model DSC822e, and evaluated with the Mettler-Toledo STAR^e^ software version 9.30 (Mettler-Toledo, OH, USA). 

The determination of transition temperatures was performed according to the following procedure: the samples of pure ionic liquids or their equimolar mixtures were cooled from to 393.15 K to 133.15 K with a relatively slow heating rate of 2 K·min^−1^ and, afterwards, they were heated from 133.15 K to 393.15 K at the same rate. Therefore, before performing the thermal analysis, the ionic liquids were dried inside the DSC by heating at T = 393.15 K for 30 min. 

To allow a good comparison with the literature of the temperatures determined in this work, it is important to understand that: the melting temperature (Tm) was taken as the onset of an endothermic peak on heating; the freezing temperature (Tf) as the onset of an exothermic peak on cooling; the glass transition temperature (Tg) as the midpoint of a small heat capacity change on heating from the amorphous glass state to a liquid state; the cold crystallization temperature (Tcc) as the onset of an exothermic peak on heating from a subcooled liquid state to a crystalline solid state; the solid-solid transition (Tss) as the onset of an exothermic or endothermic peak on heating from a crystalline solid state.

For the thermal analyses, 40 μL aluminum pans hermetically sealed with a pinhole at the top were used for the samples and a similar empty pan (also with a pinhole) was used as a reference in the furnace. The masses of the samples were between 4 and 8 mg. All the samples were weighed using a Mettler-Toledo AX-205 Delta Range balance with an uncertainty in the measurement of ±3 × 10^−4^. The calibration for temperature and heat flow was performed using the following pure substances: zinc, indium, water, and heptane. The tau lag calibration was carried out at the −20, −15, −10, 2,5, 10, and 20 K·min^−1^ rates. The standard uncertainty of the measurement of the temperature in the thermal analysis was ±1 K.

The sapphire method was chosen for the determination of the value of the molar heat capacity as a function of the temperature of the pure ionic liquids and their binary mixtures. The sapphire method consists of an initial isothermal segment for 15 min followed by a dynamic period at 20 K·min^−1^ and finally an isothermal segment for 15 min; 100 μL aluminum pans hermetically sealed with a pinhole at the top were used. The uncertainty of the measurement of the molar heat capacity was ±5%. This procedure for thermal analysis and measurement of heat capacities and the calculation of the standard uncertainties was described thoroughly in a previous publication [13].

Heat capacities were determined from different temperature ranges in the function of the melting point of the pure ionic liquid since heat capacity was only measured for the liquid range of the ionic liquids, therefore, the volume variation in the temperatures range studied can be considered negligible. For the equimolar binary mixture of two ionic liquids, the range was 258.15–348.15 K, since all mixtures studied are liquid at ambient temperature and pressure. Only data from 293.15 K to 333.15 K are presented, since, at the temperature range limits, the samples were not perfectly thermally conditioned. Therefore, the molar heat capacity for the ionic liquid 1-propyl-1-methylpiperidinium bis(trifluoromethylsulfonyl)imide was determined from 293.15–393.15 K, since this ionic liquid has a melting point at 283 K. The Cp measurements were carried out in the liquid range of the ILs, but only data from 308.15–363.15 K are presented. The Cp for the pure 1-propyl-1-methylpiperidinium trifluoromethanesulfonate ionic liquid was not determined, since this ionic liquid is solid up to a temperature of 333 K.

## 4. Conclusions

In this work, the thermal behavior and the molar heat capacities for 5 equimolar binary mixtures of ionic liquids were determined and evaluated by DSC. The results obtained in this work for the mixtures were compared with the thermal behavior and the molar heat capacities of the respective pure ionic liquids previously published by the group. Moreover, the transition temperatures for two pure piperidinium-based ionic liquids and the heat capacities for one pure piperidinium-based ionic liquid were measured in an assay.

The results show that mixing different ionic liquids leads to a new material with improved characteristics when compared to the starting components. The thermal behavior of the binary mixtures of the ionic liquids (which present melting points) demonstrated that the mixtures do not have melting points, but only present glass transition temperatures.

It was shown that the Cp values of the binary mixtures of ionic liquids are in the interval between the values of Cp for the pure ionic liquids present in the mixture. Furthermore, the trend found for the value of the heat capacities of the pure ionic liquid was as follows: piperidinium > pyrrolidinium > pyridinium > imidazolium-based ionic liquids.

## Figures and Tables

**Table 1:** Results of the thermal analysis for the pure ionic liquids and comparison with the literature ^a^.

Ionic Liquid	Tf_(L-I)_ / K	Tf_(I-II)_ / K	Tf_(II-III)_ / K	Tss_(III-II)_ / K	Tss_(II-I)_ / K	Tm_(I-L)_ / K
(1,1) PMpipNTf_2_	273					283 (286) ^b,c^
(1,1) PMpipTFO	173	183	323	173	258	323

^a^ Standard uncertainty *u*(*T*) = ±1 K; ^b^ from reference [8] at 10 K·min^−1^ rate; ^c^ from reference [9] at 10 K·min^−1^ rate.

**Table 2:** Fitting parameters for the fitting (Cp = *y_0_* + *a*T) of the experimental molar heat capacities for PMpipNTf_2_ ionic liquid.

IL	*y*_0_	*a*	R^2^
(1,1) PMpipNTf_2_	247.37	1.1235	0.9998

**Table 3:** Results of the thermal analysis for the pure ILs [10,11,12] and their mixtures ^a^.

IL/Mixture of ILs	Tg / K	Tf_(L-I)_ / K	Tf_(I-II)_ / K	Tf_(II-III)_ / K	Tcc_1_ / K	Tcc_2_ / K	Tss_(III-II)_ / K	Tss _(II-I)_ / K	Tm_(I-L)_ / K
(1,3) BMimTFO ^b^		253	258						289
(1,3) BMimDCA ^b^		181				225		266	269
(1,3) BMimNTF_2_ ^c^	184								
(1,3) BMimTFO + (1,3) BMimNTf_2_	178								
(1,3) BMimDCA + (1,3) BMimNTf_2_	181								
(1,3) BMpyTFO ^b^		255	256						
(1,3) BMpyNTf_2_^b^	188								
(1,3) BMpyTFO + (1,3) BMpyNTf_2_	188								
(1,1) BMpyrTFO ^d^		245						238	267
(1,1) BMpyrNTf_2_^d^	183				208			244	253
(1,1) BMpyrTFO + (1,1) BMpyrNTf_2_	183								
(1,1) PMpipTFO ^e^		173	183	333			173	258	333
(1,1) PMpipNTf_2_^e^		273							283
(1,1) PMpipTFO + (1,1) PMpipNTf_2_	198								

^a^ Standard uncertainty *u*(*T*) = ±1 K; ^b^ from reference [11]; ^c^ from reference [10]; ^d^ from reference [12]; ^e^ this work.

**Table 4:** Experimental heat capacities, Cp, for the pure ILs [10,11,12] and their mixtures at *T* = 323.15 K ^a^.

Ionic Liquid or Mixture	Cp/J·K^−1^·mol^−1^
(1,3) BMimTFO ^b^	456
(1,3) BMimDCA ^b^	387
(1,3) BMimNTf_2_^c^	551
(1,3) BMimTFO + (1,3) BMimNTf_2_	549
(1,3) BMimDCA + (1,3) BMimNTf_2_	516
(1,3) BMpyNTf_2_^b^	572
(1,3) BMpyTFO ^b^	461
(1,3) BMpyTFO+ (1,3) BMpy NTf_2_	563
(1,1) BMpyrNTf_2_^c^	590
(1,1) BMpyrTFO ^c^	465
(1,1) BMpyrTFO + (1,1) BMpyrNTf_2_	571
(1,1) PMpipNTf_2_^d^	610
(1,1) PMpipTFO ^d^	n.a. ^e^
(1,1) PMpipTFO + (1,1) PMpipNTf_2_	593

^a^ Standard uncertainty *u*(T) = ±5%; ^b^ from reference [11]; ^c^ from reference [10]; ^d^ from reference [12]; ^e^ This work; n.a.—not available.

**Table 5:** Fitting parameters for the fitting (Cp = *y*_0_ + *a*T *+ b*T^2^) of the experimental molar heat capacities for equimolar binary mixtures.

Binary Mixtures	*y*_0_	*a*	*b*	R^2^
(1,3) BMimTFO + (1,3) BMimNTf_2_	640.2	−1.86	0.0049	0.9999
(1,3) BMimDCA + (1,3) BMimNTf_2_	687.0	−2.18	0.0051	0.9999
(1,3) BMpyTFO + (1,3) BMpy NTf_2_	617.5 605.8 570.1	−1.71	0.0048	0.9999
(1,1) BMpyrTFO + (1,1) BMpyrNTf_2_	605.8	−1.63	0.0047	0.9999
(1,1) PMpipTFO + (1,1) PMpipNTf_2_	570.1	−1.40	0.0045	0.9999

**Table 6:** Name, abbreviation, purity, and water content of pure ionic liquids.

Name	Abbreviation	Purity ^a^ (Mole Fraction)	Water Content ^b^ (ppm)
1-butyl-3-methylimidazolium bis(trifluoromethylsulfonyl)imide	(1,3) BMimNTf_2_	98%	<100
1-butyl-3-methylimidazolium trifluoromethanesulfonate	(1,3) BMimTFO	98%	<300
1-butyl-3-methylimidazolium dycianamide	(1,3) BMimDCA	99%	<300
1-butyl-3-methylpyridinium bis(trifluoromethylsulfonyl)imide	(1,3) BMpyNTf_2_	98%	<300
1-butyl-3-methylpyridinium trifluoromethanesulfonate	(1,3) BMpyTFO	98%	<300
1-butyl-1-methylpyrridinium bis(trifluoromethylsulfonyl)imide	(1,1) BMpyrNTf_2_	99%	<100
1-butyl-1-methylpyrridinium trifluoromethanesulfonate	(1,1) BMpyrTFO	99%	<100
1-methyl-1-propylpipiridinium bis(trifluoromethylsulfonyl)imide	(1,1) PMpipNTf_2_	99%	<200
1-methyl-1-propylpipiridinium trifluoromethanesulfonate	(1,1) PMpipTFO	99%	<200

^a^ Mole fraction purity and analysis method given by the company; ^b^ standard uncertainty *u* (water content) = ±5%.

## Data Availability

Not applicable.

## Supplementary Materials

The following are available online, Table S1. Experimental molar heat capacities, Cp, as function of temperature for the (1,1) PMpipNTf_2_ ionic liquid. Table S2. Experimental molar heat capacities, Cp, as function of temperature for equimolar binary mixtures. Figure S1. Thermogram cooling the sample from 393.15 K to 133.15 K and heating from 393.15 K to 133.15 K at a rate of 2 K·min^−1^ for (a) (1,3) BMimTFO + (1,3) BMimNTf_2_; (b) (1,3) BMimDCA + (1,3) BMimNTf_2_; (c) (1,3) BMpyTFO + (1,3) BMpyNTf_2_ and (d) (1,1) BMpyrTFO + (1,1) BMpyrNTf_2_.

## Sample Availability

Samples of the compounds are not available from the authors.
