# Supplementary material for: Thermal Analysis of Binary Mixtures of Imidazolium, Pyridinium, Pyrrolidinium, and Piperidinium Ionic Liquids
Source: Molecules. 2021 Oct 22;26(21):6383. doi: 10.3390/molecules26216383 (PMC8587281; doi:10.3390/molecules26216383)
Supplement: Supplementary file 1 [file molecules-26-06383-s001.zip › molecules-1425460-supplementary.pdf]

Thermal analysis of binary mixtures of imidazolium. pyridinium. pyrrolidinium and piperidinium ionic liquids

Elena Gómez[a], Pedro Velho[a], Ángeles Domínguez[b], Eugénia A. Macedo[a,]*

[a]Associate Laboratory of Separation and Reaction Engineering – Laboratory of Catalysis and Materials (LSRE-LCM). Department of Chemical Engineering. Faculty of Engineering. University of Porto. Rua Dr. Roberto Frias. s/n. Porto 4200-465. Portugal

[b]Advanced Separation Processes Group. Department of Chemical Engineering. University of Vigo. Campus Lagoas-Marcosende. 36310 Vigo. Spain.

*Corresponding author. Tel.: +351 22508 1653; E-mail address: eamacedo@fe.up.pt

Supporting Material

Table S1. Experimental molar heat capacities. Cp. as function of temperature for the (1,1) PMpipNTf_2 ionic liquid.

Table S2. Experimental molar heat capacities. Cp. as function of temperature for equimolar binary mixtures.

Figure S1. Thermogram cooling the sample from 393.15 K to 133.15 K and heating from 393.15 K to 133.15 K at a rate of 2 K·min^{-1} for a) (1,3) BMimTFO + (1,3) BMimNTf_2; b) (1,3) BMimDCA + (1,3) BMimNTf_2; c) (1,3) BMpyTFO + (1,3) BMpy NTf_2 and d) (1,1) BMpyrTFO + (1,1) BMpyrNTf_2.

Table S1. Experimental molar heat capacities. Cp. as function of temperature for the (1,1) $PMpipNTF_2$.

T / K	Cp / $J \cdot K^{-1} \cdot mol^{-1}$	T / K	Cp / $J \cdot K^{-1} \cdot mol^{-1}$
308.15	594	339.15	628
309.15	595	340.15	630
310.15	596	341.15	631
311.15	597	342.15	632
312.15	598	343.15	633
313.15	599	344.15	634
314.15	600	345.15	635
315.15	601	346.15	636
316.15	603	347.15	637
317.15	604	348.15	639
318.15	605	349.15	640
319.15	606	350.15	641
320.15	607	351.15	642
321.15	608	352.15	643
322.15	609	353.15	644
323.15	610	354.15	645
324.15	612	355.15	646
325.15	613	356.15	648
326.15	614	357.15	649
327.15	615	358.15	650
328.15	616	359.15	651
329.15	617	360.15	652
330.15	618	361.15	653
331.15	619	362.15	654
332.15	621	363.15	655
333.15	622		
334.15	623		
335.15	624		
336.15	625		
337.15	626		
338.15	627		

Table S2. Experimental molar heat capacities. Cp. as function of temperature for the equimolar binary mixtures.

T / K	Cp / $J \cdot K^{-1} \cdot mol^{-1}$	T / K	Cp / $J \cdot K^{-1} \cdot mol^{-1}$
(1,3) BMimTFO + (1,3) $BMimNTf_2$		(1,3) BMimDCA + (1,3) $BMimNTf_2$	
293.15	514	293.15	487.09
294.15	515	294.15	487.91
295.15	516	295.15	488.74
296.15	517	296.15	489.58
297.15	518	297.15	490.42
298.15	519	298.15	491.28
299.15	520	299.15	492.15
300.15	522	300.15	493.03
301.15	523	301.15	493.92
302.15	524	302.15	494.82
303.15	525	303.15	495.73
304.15	526	304.15	496.65
305.15	527	305.15	497.58
306.15	528	306.15	498.52
307.15	529	307.15	499.47
308.15	530	308.15	500.44
309.15	532	309.15	501.41
310.15	533	310.15	502.39
311.15	534	311.15	503.38
312.15	535	312.15	504.38
313.15	536	313.15	505.39
314.15	537	314.15	506.42
315.15	539	315.15	507.45
316.15	540	316.15	508.49
317.15	541	317.15	509.54
318.15	542	318.15	510.61
319.15	544	319.15	511.68
320.15	545	320.15	512.76
321.15	546	321.15	513.86
322.15	547	322.15	514.96
323.15	549	323.15	516.07
324.15	550	324.15	517.20
325.15	551	325.15	518.33
326.15	553	326.15	519.48
327.15	554	327.15	520.63
328.15	555	328.15	521.80
329.15	557	329.15	522.97
330.15	558	330.15	524.16
331.15	559	331.15	525.35
332.15	561	332.15	526.56
333.15	562	333.15	527.78

Table S2. Experimental molar heat capacities. Cp. as function of temperature for the equimolar binary mixtures (Continuation).

T / K	Cp / J·K^{-1}·mol^{-1}	T / K	Cp / J·K^{-1}·mol^{-1}	T / K	Cp / J·K^{-1}·mol^{-1}
(1,3)BMpyTFO+(1,3)BMpyNTf$_2$		(1,1)BMpyrDCA+(1,1)BMpyrNTf$_2$		(1,1)PMpipDCA+(1,1)PMpipNTf$_2$	
293.15	526.13	293.15	532.94	293.15	550.72
294.15	527.22	294.15	534.08	294.15	551.99
295.15	528.33	295.15	535.22	295.15	553.27
296.15	529.44	296.15	536.38	296.15	554.56
297.15	530.56	297.15	537.55	297.15	555.85
298.15	531.69	298.15	538.72	298.15	557.16
299.15	532.82	299.15	539.91	299.15	558.48
300.15	533.97	300.15	541.10	300.15	559.80
301.15	535.13	301.15	542.31	301.15	561.13
302.15	536.30	302.15	543.52	302.15	562.47
303.15	537.48	303.15	544.75	303.15	563.83
304.15	538.66	304.15	545.98	304.15	565.19
305.15	539.86	305.15	547.22	305.15	566.55
306.15	541.07	306.15	548.47	306.15	567.93
307.15	542.28	307.15	549.73	307.15	569.32
308.15	543.51	308.15	551.00	308.15	570.72
309.15	544.74	309.15	552.28	309.15	572.12
310.15	545.98	310.15	553.57	310.15	573.54
311.15	547.24	311.15	554.87	311.15	574.96
312.15	548.50	312.15	556.18	312.15	576.39
313.15	549.77	313.15	557.50	313.15	577.84
314.15	551.06	314.15	558.82	314.15	579.29
315.15	552.35	315.15	560.16	315.15	580.75
316.15	553.65	316.15	561.51	316.15	582.22
317.15	554.96	317.15	562.86	317.15	583.69
318.15	556.28	318.15	564.23	318.15	585.18
319.15	557.61	319.15	565.60	319.15	586.68
320.15	558.95	320.15	566.98	320.15	588.18
321.15	560.30	321.15	568.38	321.15	589.70
322.15	561.66	322.15	569.78	322.15	591.22
323.15	563.03	323.15	571.19	323.15	592.75
324.15	564.40	324.15	572.61	324.15	594.29
325.15	565.79	325.15	574.04	325.15	595.85
326.15	567.19	326.15	575.48	326.15	597.41
327.15	568.59	327.15	576.93	327.15	598.97
328.15	570.01	328.15	578.39	328.15	600.55
329.15	571.43	329.15	579.86	329.15	602.14
330.15	572.87	330.15	581.34	330.15	603.74
331.15	574.31	331.15	582.83	331.15	605.34
332.15	575.77	332.15	584.32	332.15	606.96
333.15	577.23	333.15	585.83	333.15	608.58

a)

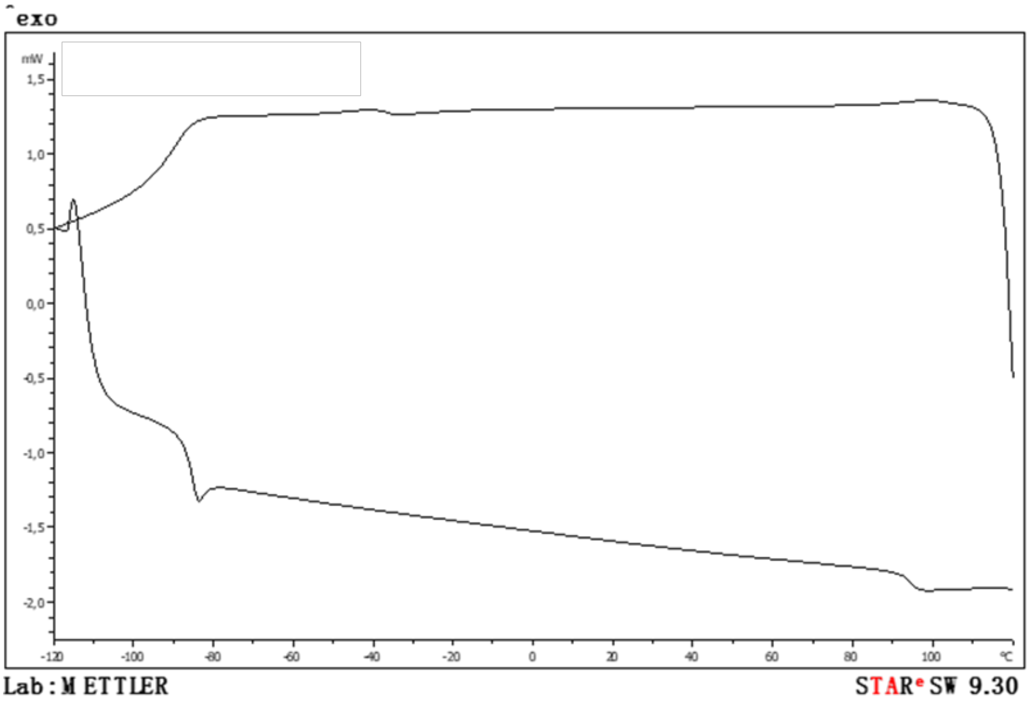
^exo
mW
1,5
1,0
0,5
0,0
-0,5
-1,0
-1,5
-2,0
-120
-100
-80
-60
-40
-20
0
20
40
60
80
100
°C
Lab: METTLER
STARe SW 9.30

b)

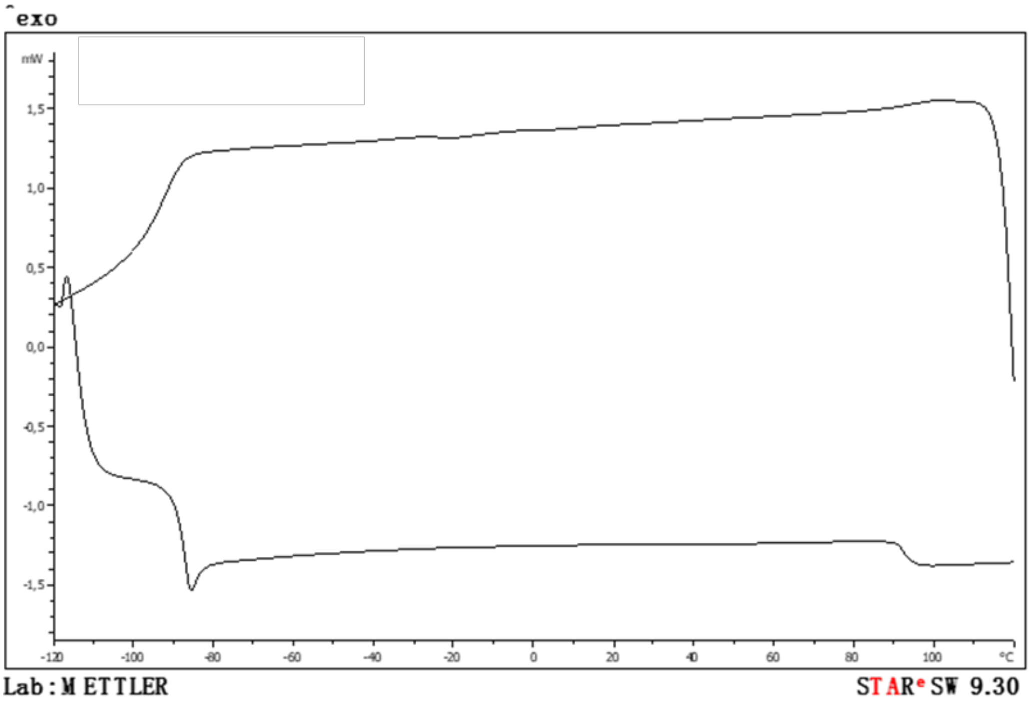
^exo
mW
1,5
1,0
0,5
0,0
-0,5
-1,0
-1,5
-120
-100
-80
-60
-40
-20
0
20
40
60
80
100
°C
Lab: METTLER
STARe SW 9.30

c)

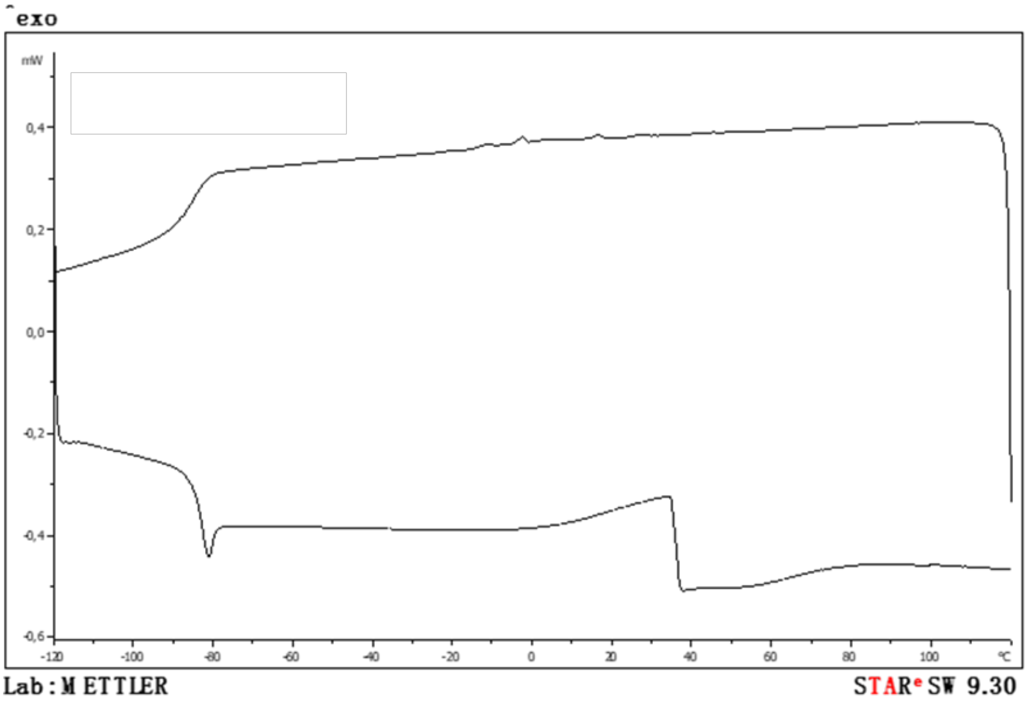

d)

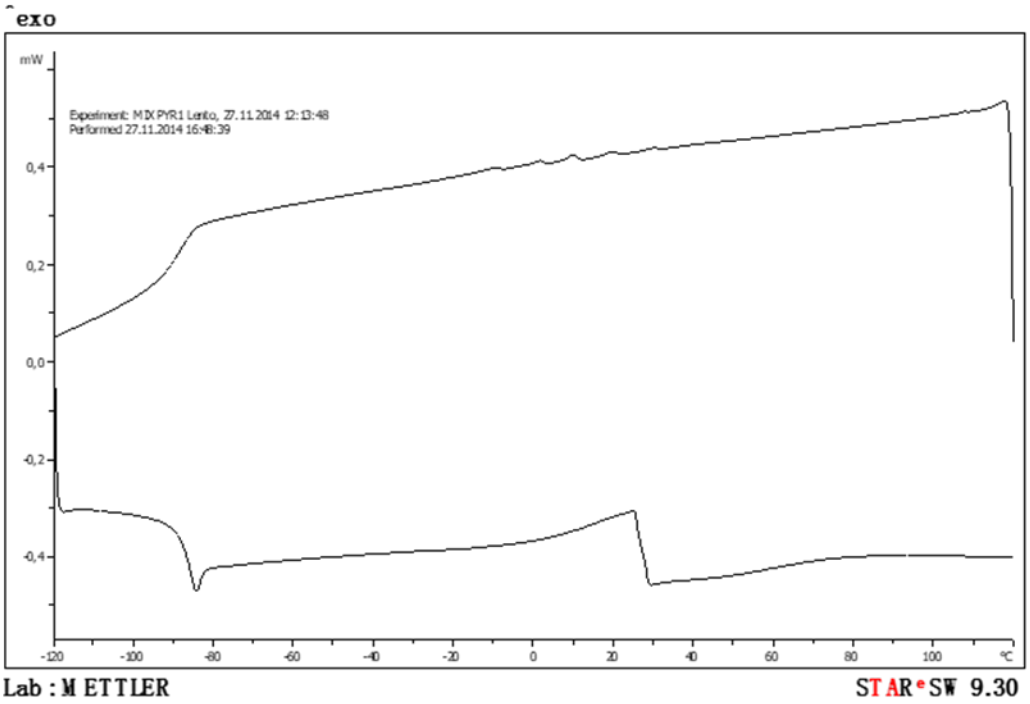

Figure S1. Thermogram cooling the sample from 393.15 K to 133.15 K and heating from 393.15 K to 133.15 K at a rate of 2 K·min^{-1} for a) (1,3) BMimTFO + (1,3) BMimNTf$_2$; b) (1,3) BMimDCA + (1,3) BMimNTf$_2$; c) (1,3) BMpyTFO + (1,3) BMpyNTf$_2$ and d) (1,1) BMpyrTFO + (1,1) BMpyrNTf$_2$.
